# Crystal structure of *tert*-butyl-*N*-phenyl­carbonitrilium tetra­chlorido­aluminate

**DOI:** 10.1107/S1600536814022028

**Published:** 2014-10-11

**Authors:** Tom van Dijk, Dirk W. Zant, Robert Wolf, Koop Lammertsma, J. Chris Slootweg

**Affiliations:** a Department of Chemistry and Pharmaceutical Sciences, VU University Amsterdam, De Boelelaan 1083, 1081 HV Amsterdam, The Netherlands; b Institute of Inorganic Chemistry, University of Regensburg, 93040 Regensburg, Germany

**Keywords:** crystal structure, nitrilium ion, tetra­chlorido­aluminate

## Abstract

In the title compound, (C_11_H_14_N)[AlCl_4_], the nitrilium (systematic name: 2,2-dimethyl-*N*-phenyl­propane­nitrilium) ion adopts a slightly distorted linear configuration [C—N C = 178.87 (16) and N C—C = 179.13 (17)°]. In the crystal, while there are no inter­molecular hydrogen bonds, pairs of nitrilium ions are linked through π–π inter­actions [inter–centroid distance = 3.8091 (13) Å].

## Abstract 2

The nitrilium cations of the title compound adopt a slightly distorted linear configuration and are linked through π–π inter­actions. The nitrilium cations and tetra­aluminate anions are arranged in alternating planes parallel to the (011) plane.

## Chemical context   

Nitrilium salts are highly electrophilic species that can be generated from imidoyl chlorides by abstracting its chloride using a Lewis acid, SbCl_5_ having been most widely applied (Meerwein, Laasch, Mersch & Nentwig, 1956 ▶; Klages & Grill, 1955 ▶; Kanemasa, 2004 ▶). Recently, we have shown that tri­methyl­silyl triflate (TMSOTf) can also be used as a Lewis acid, generating nitrilium triflates, which are excellent imine synthons in the preparation of 1,3-imino­phosphane ligands (van Dijk *et al.*, 2014 ▶). Inter­estingly, nitrilium tetra­chlorido­aluminates, which can be synthesised using the much cheaper AlCl_3_, have found little application (Meerwein, Laasch, Mersch & Spille, 1956 ▶; Al-Talib *et al.* 1992 ▶). Therefore, we also focused on these species of which the title compound is illustrative.
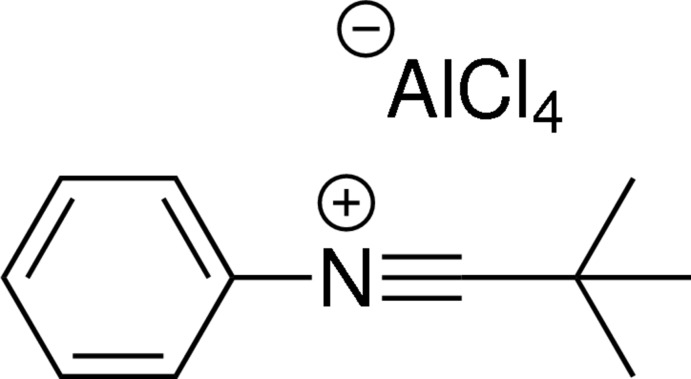


## Structural commentary   

**Figure 1:**
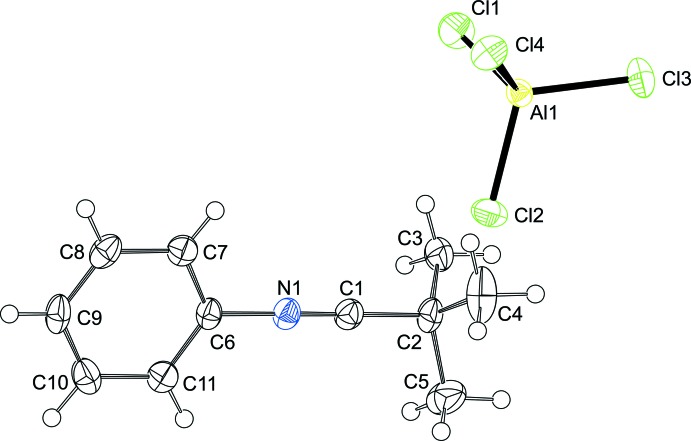
Mol­ecular structure of *tert*-butyl-*N*-phenyl­carbonitrilium tetra­chlorido­aluminate with displacement ellipsoids drawn at the 50% probability level.

The asymmetric unit of the crystal (Fig. 1 ▶) contains one nitrilium cation and one tetra­chlorido­aluminate anion, which are ion-separated. The nitrilium cation adopts a slightly distorted linear configuration [C—N C = 178.87 (16) and N C–C = 179.13 (17)°] and features an N C bond length of 1.1353 (19) Å, which is in the range of previously reported nitrilium ions (see *Database survey*). The tetra­chlorido­aluminate anion has an approximately tetra­hedral geometry and is in the range of those reported previously (Bezombes *et al.*, 2004 ▶).

## Supra­molecular features   

In the unit cell, pairs of inversion-related nitrilium cations are linked through π–π inter­actions with an inter-centroid distance of 3.8091 (13) Å. There is a plane-to-plane shift of the phenyl rings of 1.563 (3) Å. The nitrilium cations and tetra­chloridoaluminate anions are arranged in alternating planes parallel to (011).

## Database survey   

A search in the Cambridge Structural Database (Version 5.35, last update May 2014; Groom & Allen, 2014 ▶) showed five structures of nitrilium salts (Gjøystdal & Rømming, 1977 ▶; MacLaughlin *et al.*, 1983 ▶; Casey *et al.*, 1988 ▶; Bykhovskaya *et al.*, 1993 ▶, Okazaki *et al.*, 2013 ▶), and two structures of nitrilium ylides (Janulis *et al.*, 1984 ▶; Doherty *et al.*, 1999 ▶)). The title compound is very closely related to *N*-(2,6-di­methyl­phen­yl)-acetonitrilium tetra­fluorido­borate (Gjøystdal & Rømming, 1977 ▶), which has an N C bond length of 1.131 Å, and (*N*-phen­yl)(*tert*-but­yl)carbonitrilium tri­fluoro­methane­sulfonate [van Dijk *et al.*, 2014 ▶; N C bond length of 1.125 (3) Å], both of which feature similar bond lengths and angles for the nitrilium group.

## Synthesis and crystallization   

This experiment was performed under an atmosphere of dry nitro­gen using standard Schlenk-line and glovebox techniques. NMR spectra were recorded at 300 K on a Bruker Advance 500 and referenced inter­nally to residual solvent resonance of CD_2_Cl_2_, ^1^H at δ 5.32, ^13^C{^1^H} at δ 53.84. The melting point was measured in a sealed capillary on a Stuart Scientific SMP3 melting point apparatus and is uncorrected. The IR spectrum was recorded on a Shimadzu FTIR–8400S spectrophotometer. Solvents were distilled from the appropriate drying agents CaH_2_ (DCM), NaK/benzo­phenone (diethyl ether), and P_2_O_5_ (CD_2_Cl_2_), and kept under an inert atmosphere of dry nitro­gen.

The title compound was obtained as follows: to a suspension of AlCl_3_ (3.00 g, 22.4 mmol) in DCM (10 ml) cooled to 195 K, an equimolar amount of *N*-phenyl­pivalimidoyl chloride (4.38 g, 22.4 mmol) in DCM (25 ml) was added dropwise, after which the reaction mixture was warmed to room temperature and stirred for 16 h. All volatiles were removed *in vacuo*, after which the product was redissolved in DCM (60 ml), layered with diethyl ether (90 ml) and cooled to 193 K for 48 h. The analytically pure product was isolated as a grey crystalline solid (6.23 g, 18.9 mmol, 85%). Recrystallization from DCM at 278 K yielded crystals suitable for X-ray crystallography. The crystals were coated with paratone oil and mounted on a glass fibre in the cooled nitro­gen stream of the diffractometer. M.p. 411 K. ^1^H NMR (500.2 MHz, CD_2_Cl_2_): δ 7.89 [*d*, ^3^ *J*(H,H) = 7.6 Hz, 2H; *o*-Ph*H*], 7.80 [*t*, ^3^ *J*(H,H) = 7.6 Hz, 1H; *p*-Ph*H*], 7.66 [*t*, ^3^ *J*(H,H) = 7.6 Hz, 2H; *m*-Ph*H*], 1.84 [*s*, 9H; C(C*H*_3_)_3_]. ^13^C{^1^H} NMR (125.8 MHz, CD_2_Cl_2_): δ 135.1 (*s*; *p*-Ph*C*), 131.0 (*s*; *m*-Ph*C*), 128.7 (*s*; *o*-Ph*C*), 121.1 [*t*, ^1^ *J*(C,N) = 42.7 Hz; N *C*], 120.7 [*t*, ^1^ *J*(C,N) = 14.2 Hz; *ipso*-Ph*C*], 31.7 [*s*; *C*(CH_3_)_3_], 27.3 [*s*; C(*C*H_3_)_3]_. IR: 3065 (*w*), 2990 (*w*), 1692 (*w*), 1611 (*w*), 1588 (*w*), 1483 (*w*), 1474 (*m*), 1456 (*m*), 1445 (*w*), 1373 (*w*), 1296 (*w*), 1238 (*w*), 1198 (*w*), 1186 (*w*), 1161 (*w*), 1028 (*w*), 1005 (*w*), 939 (*w*), 928 (*w*), 876 (*w*), 845 (*w*), 781 (*w*), 758 (*s*), 692 (*w*), 677 (*m*), 669 (*w*), 652 (*w*).

## Refinement   

Crystal data, data collection and structure refinement details are summarized in Table 1 ▶.

## Supplementary Material

Crystal structure: contains datablock(s) I, global. DOI: 10.1107/S1600536814022028/kj2242sup1.cif

Structure factors: contains datablock(s) I. DOI: 10.1107/S1600536814022028/kj2242Isup2.hkl

CCDC reference: 1027790

Additional supporting information:  crystallographic information; 3D view; checkCIF report

## Figures and Tables

**Table 1:** Experimental details

Crystal data	
Chemical formula	(C_11_H_14_N)[AlCl_4_]
*M*_r_	329.03
Crystal system, space group	Monoclinic, *P*2_1_/*c*
Temperature (K)	153
*a*, *b*, *c* ()	6.4531(6), 13.6967(13), 17.9352(17)
()	93.636(1)
*V* (^3^)	1582.0(3)
*Z*	4
Radiation type	Mo *K*
(mm^1^)	0.78
Crystal size (mm)	0.16 0.05 0.03

Data collection	
Diffractometer	Bruker APEXII
Absorption correction	Multi-scan (*SADABS*; Bruker, 2007 ▶)
*T*_min_, *T*_max_	0.885, 0.977
No. of measured, independent and observed [*I* > 2(*I*)] reflections	16388, 4063, 3294
*R*_int_	0.035
(sin /)_max_ (^1^)	0.675

Refinement	
*R*[*F* ^2^ > 2(*F* ^2^)], *wR*(*F* ^2^), *S*	0.030, 0.081, 1.03
No. of reflections	4063
No. of parameters	210
H-atom treatment	All H-atom parameters refined
_max_, _min_ (e ^3^)	0.35, 0.26

Computer programs: *APEX2* and *SAINT-Plus* (Bruker, 2007 ▶), *SHELXS97* and *SHELXL97* (Sheldrick, 2008 ▶), *PLATON* (Spek, 2009 ▶), *WinGX* (Farrugia, 2012 ▶) and *OLEX2* (Dolomanov *et al.*, 2009 ▶).

## supplementary crystallographic information

### Crystal data

**Table:**

(C_11_H_14_N)[AlCl_4_]	*F*(000) = 672
*M**_r_* = 329.03	*D*_x_ = 1.381 Mg m^−^^3^
Monoclinic, *P*2_1_/*c*	Melting point: 411 K
Hall symbol: -P 2ybc	Mo *K*α radiation, λ = 0.71073 Å
*a* = 6.4531 (6) Å	Cell parameters from 5175 reflections
*b* = 13.6967 (13) Å	θ = 2.3–28.5°
*c* = 17.9352 (17) Å	µ = 0.78 mm^−^^1^
β = 93.636 (1)°	*T* = 153 K
*V* = 1582.0 (3) Å^3^	Needle, colorless
*Z* = 4	0.16 × 0.05 × 0.03 mm

### Data collection

**Table:**

Bruker APEXII diffractometer	4063 independent reflections
Radiation source: rotating anode	3294 reflections with *I* > 2σ(*I*)
Graphite monochromator	*R*_int_ = 0.035
Detector resolution: 80 pixels mm^-1^	θ_max_ = 28.7°, θ_min_ = 1.9°
ω and Phi scans	*h* = −8→8
Absorption correction: multi-scan (*SADABS*; Bruker, 2007)	*k* = −18→18
*T*_min_ = 0.885, *T*_max_ = 0.977	*l* = −24→24
16388 measured reflections	

### Refinement

**Table:**

Refinement on *F*^2^	Primary atom site location: structure-invariant direct methods
Least-squares matrix: full	Secondary atom site location: difference Fourier map
*R*[*F*^2^ > 2σ(*F*^2^)] = 0.030	Hydrogen site location: inferred from neighbouring sites
*wR*(*F*^2^) = 0.081	All H-atom parameters refined
*S* = 1.03	*w* = 1/[σ^2^(*F*_o_^2^) + (0.0425*P*)^2^ + 0.1953*P*] where *P* = (*F*_o_^2^ + 2*F*_c_^2^)/3
4063 reflections	(Δ/σ)_max_ = 0.001
210 parameters	Δρ_max_ = 0.35 e Å^−^^3^
0 restraints	Δρ_min_ = −0.26 e Å^−^^3^

### Special details

**Table:**

Experimental. Corrections were done with the *SADABS* program, utilizing the none merged raw data obtained from the integration process. Integration and final cell refinement were done with *SAINT*.*SADABS* reports ratio of Tmin/Tmax = 0.797049
Geometry. All e.s.d.'s (except the e.s.d. in the dihedral angle between two l.s. planes) are estimated using the full covariance matrix. The cell e.s.d.'s are taken into account individually in the estimation of e.s.d.'s in distances, angles and torsion angles; correlations between e.s.d.'s in cell parameters are only used when they are defined by crystal symmetry. An approximate (isotropic) treatment of cell e.s.d.'s is used for estimating e.s.d.'s involving l.s. planes.
Refinement. Refinement of *F*^2^ against ALL reflections. The weighted *R*-factor *wR* and goodness of fit *S* are based on *F*^2^, conventional *R*-factors *R* are based on *F*, with *F* set to zero for negative *F*^2^. The threshold expression of *F*^2^ > σ(*F*^2^) is used only for calculating *R*-factors(gt) *etc*. and is not relevant to the choice of reflections for refinement. *R*-factors based on *F*^2^ are statistically about twice as large as those based on *F*, and *R*- factors based on ALL data will be even larger.

### Fractional atomic coordinates and isotropic or equivalent isotropic displacement parameters (Å^2^)

**Table:**

	*x*	*y*	*z*	*U*_iso_*/*U*_eq_	
Al1	0.96022 (7)	0.22733 (3)	0.35138 (2)	0.02252 (11)	
C1	0.5146 (2)	0.96503 (11)	0.27377 (8)	0.0280 (3)	
C2	0.5280 (3)	0.91716 (11)	0.34748 (8)	0.0301 (3)	
C3	0.6685 (3)	0.82750 (13)	0.34364 (10)	0.0333 (3)	
C4	0.6215 (5)	0.99100 (15)	0.40414 (11)	0.0544 (6)	
C5	0.3056 (3)	0.88817 (17)	0.36455 (14)	0.0492 (5)	
C6	0.4893 (2)	1.04868 (10)	0.14690 (8)	0.0246 (3)	
C7	0.6619 (3)	1.09623 (11)	0.12260 (9)	0.0287 (3)	
C8	0.6442 (3)	1.13970 (11)	0.05267 (9)	0.0321 (3)	
C9	0.4587 (3)	1.13540 (11)	0.00948 (9)	0.0329 (4)	
C10	0.2892 (3)	1.08788 (12)	0.03538 (9)	0.0337 (4)	
C11	0.3023 (3)	1.04317 (11)	0.10465 (9)	0.0290 (3)	
Cl1	1.07491 (7)	0.12249 (3)	0.27615 (2)	0.03826 (11)	
Cl2	0.63285 (6)	0.24128 (3)	0.33003 (2)	0.03784 (11)	
Cl3	1.03104 (7)	0.17986 (3)	0.46328 (2)	0.03810 (11)	
Cl4	1.09572 (6)	0.36715 (3)	0.33438 (2)	0.03407 (11)	
N1	0.5044 (2)	1.00304 (9)	0.21740 (7)	0.0270 (3)	
H5	0.678 (3)	0.7947 (13)	0.3913 (11)	0.032 (4)*	
H6	0.803 (3)	0.8453 (14)	0.3297 (11)	0.042 (5)*	
H12	0.449 (3)	1.1638 (13)	−0.0347 (10)	0.031 (4)*	
H14	0.781 (3)	1.0977 (13)	0.1524 (10)	0.037 (5)*	
H10	0.189 (3)	1.0117 (13)	0.1246 (10)	0.033 (5)*	
H4	0.615 (3)	0.7821 (17)	0.3065 (13)	0.059 (6)*	
H13	0.760 (3)	1.1708 (14)	0.0343 (10)	0.037 (5)*	
H3	0.311 (3)	0.8571 (16)	0.4147 (13)	0.059 (6)*	
H8	0.635 (4)	0.9609 (17)	0.4530 (14)	0.067 (7)*	
H11	0.165 (3)	1.0868 (15)	0.0039 (12)	0.054 (6)*	
H7	0.539 (3)	1.0444 (18)	0.4074 (12)	0.055 (6)*	
H1	0.229 (4)	0.9457 (17)	0.3674 (13)	0.061 (7)*	
H2	0.243 (4)	0.8431 (19)	0.3264 (15)	0.074 (8)*	
H9	0.763 (4)	1.0135 (19)	0.3925 (15)	0.080 (8)*	

### Atomic displacement parameters (Å^2^)

**Table:**

	*U*^11^	*U*^22^	*U*^33^	*U*^12^	*U*^13^	*U*^23^
Al1	0.0237 (2)	0.0221 (2)	0.0215 (2)	0.00139 (16)	−0.00057 (16)	−0.00156 (15)
C1	0.0313 (8)	0.0270 (7)	0.0263 (8)	0.0037 (6)	0.0048 (6)	0.0011 (6)
C2	0.0392 (9)	0.0296 (8)	0.0220 (7)	0.0008 (6)	0.0055 (6)	0.0068 (6)
C3	0.0368 (9)	0.0350 (8)	0.0279 (8)	0.0042 (7)	0.0003 (7)	0.0072 (7)
C4	0.100 (2)	0.0342 (10)	0.0280 (10)	−0.0045 (11)	−0.0013 (10)	0.0001 (8)
C5	0.0420 (11)	0.0531 (12)	0.0542 (13)	0.0093 (9)	0.0179 (9)	0.0245 (10)
C6	0.0331 (8)	0.0202 (6)	0.0208 (7)	0.0057 (5)	0.0036 (6)	0.0021 (5)
C7	0.0307 (8)	0.0265 (7)	0.0291 (8)	0.0033 (6)	0.0034 (6)	0.0006 (6)
C8	0.0401 (9)	0.0251 (7)	0.0323 (8)	0.0022 (6)	0.0132 (7)	0.0041 (6)
C9	0.0524 (10)	0.0266 (8)	0.0201 (7)	0.0119 (7)	0.0052 (7)	0.0043 (6)
C10	0.0408 (9)	0.0320 (8)	0.0273 (8)	0.0093 (7)	−0.0054 (7)	−0.0009 (6)
C11	0.0319 (8)	0.0253 (7)	0.0300 (8)	0.0026 (6)	0.0040 (6)	0.0008 (6)
Cl1	0.0421 (2)	0.0355 (2)	0.0370 (2)	0.00888 (16)	0.00073 (17)	−0.01397 (16)
Cl2	0.02347 (19)	0.0465 (2)	0.0431 (2)	0.00328 (15)	−0.00125 (16)	0.00606 (17)
Cl3	0.0468 (2)	0.0410 (2)	0.02528 (19)	−0.00252 (17)	−0.00698 (16)	0.00525 (16)
Cl4	0.0377 (2)	0.02523 (18)	0.0400 (2)	−0.00444 (15)	0.00856 (17)	−0.00103 (15)
N1	0.0331 (7)	0.0245 (6)	0.0238 (6)	0.0057 (5)	0.0047 (5)	0.0024 (5)

### Geometric parameters (Å, º)

**Table:**

Al1—Cl3	2.1315 (6)	C2—C5	1.539 (3)
Al1—Cl2	2.1316 (6)	C6—C7	1.384 (2)
Al1—Cl1	2.1347 (6)	C6—C11	1.386 (2)
Al1—Cl4	2.1351 (6)	C6—N1	1.4084 (18)
C1—N1	1.1353 (19)	C7—C8	1.386 (2)
C1—C2	1.473 (2)	C8—C9	1.385 (2)
C2—C4	1.530 (3)	C9—C10	1.378 (3)
C2—C3	1.531 (2)	C10—C11	1.383 (2)
			
Cl3—Al1—Cl2	110.32 (2)	C4—C2—C5	111.84 (18)
Cl3—Al1—Cl1	109.13 (3)	C3—C2—C5	111.39 (15)
Cl2—Al1—Cl1	109.04 (2)	C7—C6—C11	122.93 (14)
Cl3—Al1—Cl4	110.05 (2)	C7—C6—N1	118.70 (14)
Cl2—Al1—Cl4	107.71 (2)	C11—C6—N1	118.36 (13)
Cl1—Al1—Cl4	110.58 (3)	C6—C7—C8	117.71 (15)
N1—C1—C2	179.13 (17)	C9—C8—C7	120.42 (16)
C1—C2—C4	107.46 (14)	C10—C9—C8	120.51 (15)
C1—C2—C3	108.54 (13)	C9—C10—C11	120.48 (16)
C4—C2—C3	110.50 (17)	C10—C11—C6	117.95 (15)
C1—C2—C5	106.91 (15)	C1—N1—C6	178.87 (16)

## References

[bb1] Al-Talib, M., Jochims, J. C., Hamed, A., Wang, Q. & Ismail, A. E.-H. (1992). *Synthesis*, pp. 697–701.

[bb2] Bezombes, J.-P., Borisenko, K. B., Hitchcock, P. B., Lappert, M. F., Nycz, J. E., Rankin, D. W. H. & Robertson, H. E. (2004). *Dalton Trans.* pp. 1980–1988. 10.1039/b402926g 15252585

[bb3] Bruker (2007). *APEX2*, *SAINT-Plus* and *SADABS*. Bruker AXS Inc., Madison, Wisconsin, USA.

[bb4] Bykhovskaya, O. V., Aladzheva, I. M., Petrovskii, P. V., Antipin, M. Y., Struchkov, Y. T., Mastryukova, T. A. & Kabachnik, M. I. (1993). *Mendeleev Commun.* **3**, 200–202.

[bb5] Casey, C. P., Crocker, M., Niccolai, G. P., Fagan, P. J. & Konings, M. S. (1988). *J. Am. Chem. Soc.* **110**, 6070–6076. 10.1021/ja00226a023 22148783

[bb6] Dijk, T. van, Burck, S., Rong, M. K., Rosenthal, A. J., Nieger, M., Slootweg, J. C. & Lammertsma, K. (2014). *Angew. Chem. Int. Ed.* **53**, 9068–9071. 10.1002/anie.201405027 24975733

[bb7] Doherty, S., Hogarth, G., Waugh, M., Scanlan, T. H., Clegg, W. & Elsegood, M. R. J. (1999). *Organometallics*, **18**, 3178–3186.

[bb8] Dolomanov, O. V., Bourhis, L. J., Gildea, R. J., Howard, J. A. K. & Puschmann, H. (2009). *J. Appl. Cryst.* **42**, 339–341.

[bb9] Farrugia, L. J. (2012). *J. Appl. Cryst.* **45**, 849–854.

[bb10] Gjøystdal, A. K. & Rømming, C. (1977). *Acta Chem. Scand. Ser. B*, **31**, 56–62.

[bb11] Groom, C. R. & Allen, F. H. (2014). *Angew. Chem. Int. Ed.* **53**, 662–671. 10.1002/anie.201306438 24382699

[bb12] Janulis, E. P., Wilson, S. R. & Arduengo, A. J. (1984). *Tetrahedron Lett.* **25**, 405–408.

[bb13] Kanemasa, S. (2004). In *Science of Synthesis*, Vol. 19, edited by S.-I. Murahashi, pp. 53–63. Stuttgart: Thieme Verlag.

[bb14] Klages, F. & Grill, W. (1955). *Justus Liebigs Ann. Chem.* **594**, 21–32.

[bb15] MacLaughlin, S. A., Johnson, J. P., Taylor, N. J., Carty, A. J. & Sappa, E. (1983). *Organometallics*, **2**, 352–355.

[bb16] Meerwein, H., Laasch, P., Mersch, R. & Nentwig, J. (1956). *Chem. Ber.* **89**, 224–238.

[bb17] Meerwein, H., Laasch, P., Mersch, R. & Spille, J. (1956). *Chem. Ber.* **89**, 209–224.

[bb18] Okazaki, M., Taniwaki, W., Miyagi, K., Takano, M., Kaneko, S. & Ozawa, F. (2013). *Organometallics*, **32**, 1951–1957.

[bb19] Sheldrick, G. M. (2008). *Acta Cryst.* A**64**, 112–122. 10.1107/S0108767307043930 18156677

[bb20] Spek, A. L. (2009). *Acta Cryst.* D**65**, 148–155. 10.1107/S090744490804362X PMC2631630 19171970

